# More about Human and Bovine Tuberculosis

**Published:** 1909-02-06

**Authors:** 


### The Hospital

A JOURNAL OF

The Medical Sciences and Hospital Administration.

NEW SERIES. No. 101, VOL. IV. [No. 1173, VOL. XLV.] SATURDAY, FEBRUARY 6, 1909.

### MORE ABOUT HUMAN AND BOVINE TUBERCULOSIS.

THE Royal Commission appointed to investigate the relations between human and bovine tuberculosis has issued its third interim report, and thus brings us back to a subject which, though it has received frequent notice in these columns, amply deserves all the publicity which can be given to it. It may be of service briefly to review the events which led up to the appointment of the Commission and to the reopening of a matter for some time considered to be closed. For some years subsequent to Koch's discovery of the tubercle bacillus, the unity—or, at least, the reciprocal infectivity—of the human and bovine types of the disease was regarded as assured, and many steps were inaugurated by health authorities with a view to preventing the contamination of milk by the bacillus of bovine tuberculosis. But at the International Congress on Tuberculosis, held in London in 1900, the world of science was startled by a pronouncement, on the part of the discoverer of the bacillus himself, that the bovine and the human bacillus respectively are essentially distinct. He maintained that the bovine bacillus cannot, except on the rarest occasions, produce the disease in man, and that, even when it does so, the disease is local and not progressive, the same being true of the human bacillus in relation to the ox. Scientists were disturbed to think they had been nursing a delusion, while the manifold interests involved in the supply of dairy produce immediately took up arms against the costly and, upon this showing, unnecessary restrictions which had been put upon them. The gravity of the issues raised was reflected by the prompt appointment, in August 1901, of a Royal Commission, to inquire and report (1) whether the disease in animals and man is one and the same; (2) whether animals and man can be reciprocally infected with it; (3) under what conditions, if at all, the transmission of the disease from animals to man takes place, and what are the circumstances favourable or unfavourable to such transmission. The Commission was a strong one. Its chairman was the late Sir Michael Foster, and its members Professor Sims Woodhead, Professor Sidney Martin, Sir John McFadyean, and Sir Rubert Boyce. Upon the death of Sir Michael Foster the chair of the Commission was taken by Sir William Power, late medical officer to the Local Government Board. This body has issued three interim reports, including the one just published, and their findings may be succinctly stated as follows: The bacilli associated with human tuberculosis fall into two categories. Of these, one—called the "human" type, because it occurs in the majority of human examples of the disease—produces limited and non-progressive lesions in oxen, rabbits, pigs, and goats. But in guinea-pigs, monkeys, and anthropoid apes it results in a generalised tuberculosis. To this "human" type therefore Koch's dictum applies. But in a large group of cases, including particularly the primary tuberculous lesions of cervical and abdominal glands, the causal bacillus is absolutely indistinguishable, both culturally and morphologically, from that habitually associated with bovine tuberculosis. This, therefore, is called the "bovine" type. Injected into bovines it produces a generalised tuberculosis identical, both clinically and pathologically, with that produced by the injection into these animals of bacilli of known bovine origin. In anthropoid apes bacilli of the "bovine" type, whether of human or of bovine origin, alike give rise to generalised, progressive, and rapidly fatal tuberculosis.

It is clear then that the first two of the questions set to the Commission are conclusively answered. Although it is possible to distinguish a "bovine" and a "human" type among the bacilli responsible for human tuberculosis, these two must be considered mere variants. For in cultures all gradations are met with between the two types, while the less virulent "human" type can, by passage through a series of calves, be converted into a strain capable of producing in bovines a generalised tuberculosis such as characterises the "bovine" bacillus proper. Thus one must say that the disease in animals and man *is* one and the same, and that man and animals *can* be reciprocally affected with it. The third interim report is concerned with some important aspects of the third term of reference—that, namely, which deals with the conditions under which transmission from animals to man takes place, and the circumstances which are favourable to such transmission. Tuberculosis of the udder is fairly common in cows, and the milk of such cows always contains tubercle bacilli. But it was undecided whether tuberculous cows gave tuberculous milk in the absence of manifest lesions of the udder. Six cows were dealt with, five of which had normal udders while that of the sixth was affected, but so slightly that the lesion could not be detected during life. Three of the cows were apparently healthy, the fact that they were tuberculous being demonstrated only by their reaction to tuberculin; while the other three showed definite clinical signs of the disease. The milk of those apparently healthy failed to cause tuberculosis when injected into guinea-pigs, while that of the three which showed clinical evidences of the disease produced tuberculosis, though not invariably. Thus infection of the udder is not a necessary element in the production of tuberculous milk by tuberculous cows. Moreover, the fæces of five out of the six animals were found to contain living and virulent tubercle bacilli capable of producing tuberculosis in guinea-pigs and swine. The bearing of this item is of the highest significance, in view of the indifferent cleansing to which the udders and adjoining parts of milch-cows are commonly subjected, and the ease with which milk may become contaminated by fæcal products unless the greatest care is observed. Further, since pigs may be made tuberculous by being fed with tuberculous milk, the practice of feeding pigs with the milk from ailing cows ceases to be justifiable, both from the hygienic and the economical point of view. Altogether, the Royal Commission has already earned the thanks of the public, and it is not likely that future protestations by the eminent discoverer of the tubercle bacillus will alter the general opinion that in this particular business he has been seriously mistaken.

